# Psychedelic-induced mystical experiences: An interdisciplinary discussion and critique

**DOI:** 10.3389/fpsyt.2023.1077311

**Published:** 2023-04-05

**Authors:** Sharday Mosurinjohn, Leor Roseman, Manesh Girn

**Affiliations:** ^1^School of Religion, Queen’s University, Kingston, ON, Canada; ^2^Department of Brain Sciences, Centre for Psychedelic Research, Imperial College London, London, United Kingdom; ^3^Department of Neurology and Neurosurgery, Montreal Neurological Institute, McGill University, Montreal, QC, Canada

**Keywords:** psychedelics, mystical experience, mystical experience questionnaire, psilocybin, religious studies

## Abstract

Contemporary research on serotonergic psychedelic compounds has been rife with references to so-called ‘mystical’ subjective effects. Several psychometric assessments have been used to assess such effects, and clinical studies have found quantitative associations between ‘mystical experiences’ and positive mental health outcomes. The nascent study of psychedelic-induced mystical experiences, however, has only minimally intersected with relevant contemporary scholarship from disciplines within the social sciences and humanities, such as religious studies and anthropology. Viewed from the perspective of these disciplines—which feature rich historical and cultural literatures on mysticism, religion, and related topics—‘mysticism’ as used in psychedelic research is fraught with limitations and intrinsic biases that are seldom acknowledged. Most notably, existing operationalizations of mystical experiences in psychedelic science fail to historicize the concept and therefore fail to acknowledge its perennialist and specifically Christian bias. Here, we trace the historical genesis of the mystical in psychedelic research in order to illuminate such biases, and also offer suggestions toward more nuanced and culturally-sensitive operationalizations of this phenomenon. In addition, we argue for the value of, and outline, complementary ‘non-mystical’ approaches to understanding putative mystical-type phenomena that may help facilitate empirical investigation and create linkages to existing neuro-psychological constructs. It is our hope that the present paper helps build interdisciplinary bridges that motivate fruitful paths toward stronger theoretical and empirical approaches in the study of psychedelic-induced mystical experiences.

## 1. Introduction

Psychedelics have re-emerged as compounds of scientific and clinical interest. Preliminary clinical trials with classic psychedelics have indicated the potential for transdiagnostic efficacy spanning the treatment of depression, end-of-life distress, tobacco addiction, alcoholism, and obsessive–compulsive disorder (1–9). Within the current wave of human psychedelic research, the majority of human studies have been with psilocybin (the pro-drug of psilocin, the primary psychoactive compound in so-called ‘magic mushrooms’), with additional trials completed and underway with other ‘classic’ psychedelics such as LSD and DMT. Research with other quasi-psychedelics such as MDMA and ketamine, is further along, with ketamine licensed as a medical intervention and MDMA-assisted therapy on track for this in coming years. In addition, investigations in healthy subjects have examined psychedelics’ potential for improving well-being and enhancing creativity (10–17).

One phenomenon that has garnered a particularly large amount of attention in contemporary psychedelic research is the so-called ‘mystical experience’ (18–21). Psychometric assessments of mystical experiences have provided quantitative evidence for mystical-type phenomenology in the psychedelic experience, and findings have further indicated that such phenomenology may be central to the therapeutic action of these compounds—potentially mediating or moderating lasting symptom reductions in patients and improvements in well-being in healthy individuals (20, 22–25). The prevalence of constructs pertaining to ‘mysticism’ in scientific discourse is salient and interesting, given its connotations of spiritual and metaphysical concepts that are typically construed as outside the domain of science (26, 27). Typically, the response to this by researchers in favor of employing the language of ‘mysticism’ is that their reference to such concepts is independent of metaphysical claims or religious suppositions (27, 28). However, although psychedelic scientists may believe themselves to be avoiding any theological, supernatural, or metaphysical positions (and therefore employing ‘mysticism’ concepts differently than many study participants, patients, and press), this is often not made explicit. In fact, when defining ‘mysticism’, papers often explicitly invoke religious or religion-related concepts in the same breath—for instance, Barrett and Griffiths (19) lead their section “What Are Mystical Experiences?” with the following:

“[Mystical experiences are] those peculiar states of consciousness in which the individual discovers himself to be one continuous process with God, with the Universe, with the Ground of Being, or whatever name he may use by cultural conditioning or personal preference for the ultimate and eternal reality”.

When researchers use this label of ‘mysticism’, and especially when they pair it with theological discourses, God-talk, and reference to religions, they imply that the concept *does* have something to do with theological, supernatural, or metaphysical matters. Of course, other labels for similar psychedelic experiences—like ‘ego dissolution’ or ‘oceanic boundlessness’—carry their own religio-cultural baggage and connotations, and, indeed, all of these could and should also be historicized and critiqued (28). In the present paper, we have chosen to focus on the constructs of mysticism and the so-called mystical experience for three primary reasons: one, the putative ability for psychedelics to induce mystical experiences has received a disproportionate amount of attention both within the academic literature and culture at large; two ‘mysticism’ is closely linked to alternative concepts such as ‘ego dissolution’, ‘connectedness’, ‘awe’, and ‘oceanic boundlessness’; and three, mysticism arguably connotes a metaphysics that is intertwined with religious/theological and supernatural suppositions, and therefore may appear to clash with physicalist/scientific materialistic assumptions implicit in scientific research to a greater degree than alternative similar concepts. Collectively, these characteristics of the mystical experience as construed in psychedelic research render it a central and loaded concept which presents itself as particularly important to critique. It may still be objected that the construct indexed by the Mystical Experience Questionnaire (MEQ) and the constructs indexed by similar measures are so highly inter-correlated that they are essentially measuring the same thing, and thus, the choice of the label we use for them, and which measure we choose to deploy, is simply down to personal preferences. Our point, however, is that the importation of a culturally-loaded concept (like ‘mysticism’) into scientific practice can perpetuate its associated values and biases if not appropriately examined and critiqued.

We presently define mysticism broadly as the practice of techniques that elicit experiences which are construed as enabling access to metaphysical insight based in self-transcendence and/or extrasensory perception. The term ‘mysticism’ is derived from the Greek term *mystikos*, referring to the mystery-cults of the ancient Mediterranean world. These mystery cults were centered on secret initiatory rituals that were aimed at leading participants into the awareness of a higher reality (29). It is worth noting that the experiences thereby facilitated were understood to be important and valuable because they revealed insights and esoteric knowledge into the nature of reality, not because participants had experiences they deemed personally meaningful. Indeed, the ancient world and its mystery cults had no comparable notion to our modern Western concept of subjectivity, let alone an interest in cultivating something like personal religious experience (29). Contemporary religious studies thus understands mysticism as highly dependent on the context in which the concept appears. For religion scholars, the interpretive task with mysticism is investigating how, for instance, the mysticism of the ancient cult of Isis meant something different to the mysticism of Medieval Jewish scholars, just as the mysticism we find in the psychedelic discourse today means something different still.

Viewed through a religious studies lens, contemporary conceptions of psychedelic-induced mystical experiences contain inherent perennialist assumptions that are particularly vulnerable to critique, and, furthermore, have more to do with modern Christian notions of mysticism than is typically appreciated (see (27) and (30) for valuable complementary discussions). Our goal in the present paper is to highlight how assessments and conceptualizations of mystical experiences in the context of contemporary psychedelic science have, to date, imported Christian perennialist metaphysical assumptions that thereby limit the scope, nuance, and cross-cultural sensitivity of such investigations. Importantly, we are not advising that psychedelic science jettisons all mention of ‘mystical experiences’, but, rather, that greater explication of the limitations and biases of past work is required to valuably advance upon and refine existing approaches. In other words, we believe that discussions of mystical experience indeed have a place in psychedelic research, but that this field can do better in acknowledging and interfacing with relevant interdisciplinary critical work.

In the present paper, we provide a comprehensive, critical, and forward-looking discussion of assessments and conceptualizations of ‘mystical’ experiences in the context of psychedelic research. Our approach is explicitly interdisciplinary, and we seek to create the groundwork for bridges between relevant scholarship in the social sciences and humanities and the scientific investigation of psychedelic-induced ‘mystical’ experiences. We argue that such bridges are essential to advance this research area’s conceptual rigor and contextual inclusivity. In service of this argument, we begin by discussing the historical development of research on psychedelic-induced mystical experiences, highlighting its cultural biases and limitations. Next, we provide an overview of psychometric assessments of mystical experiences and a discussion of their limitations. Finally, we offer suggestions for ‘next-generation’ assessments and refined conceptualizations of mystical experiences. These proposed ‘next-generation’ assessments will draw from interdisciplinary scholarship and can contribute to the mutual enrichment of both scientific-medical and social sciences-humanities approaches to studying psychedelic neurobiology and phenomenology.

## 2. Historical overview

### 2.1. Historical reasons for a lack of interdisciplinary dialogue in research on psychedelic-induced mystical experiences

As a result of an interdisciplinary history involving some major overlaps and intentional distancing, contemporary psychedelic science has not benefited from scholarship in social sciences and humanities—disciplines that are responsible for the historical and cultural work focused on ‘mystical experience’, among other things. The following section highlights the threaded roots of psychedelic science in the study of psychology and religion, and their historical untethering which has ultimately resulted in the contemporary moment.

Despite having shared origins, perhaps best exemplified in the work of one of the founding figures of psychology, William James, whose theoretical interests spanned topics ranging from every day human perception, mystical states, and paranormal séance work, it was during the twentieth century that psychology and religion went their separate ways. In response to the trauma of WWII and influenced by new research in cultural anthropology, the contemporary academic study of religion began shedding its esoteric and theological roots in response to the growth of the ‘social sciences.’ Whereas theological study takes religion to be *sui generis* and divinely given—thus taking a ‘faith’ commitment to a particular religious worldview as a premise—religious studies started reconsidering religion as a human-made category that could be analyzed with social scientific methods and the tools of philosophical critique.

During this same period, the field of psychology came to be dominated by psychoanalysis and behaviorism, the latter of which treated the human being as a kind of machine (29), a very different take from its original interest in metaphysical inquiries about the human psyche or soul (in the original Greek). Much like the separation of the study of religion from theology, it was with these developments that “academic psychology distanced itself from its deep historical involvement with [a form of religion called] Western esotericism” (29). Broadly, ‘esotericism’ refers to the history of practices rejected by both mainstream science and the ‘world religions’, and which can be roughly captured in the three categories of: magic, alchemy, and astrology. Before this time, esoteric ideas like “mesmerism and somnambulism developed in straight lines towards experimental psychology and psychiatry as practiced in the decades around 1900” (29). With behaviorism, especially (for psychoanalysis retained many esoteric concepts) academic psychology was effectively shorn of *most* untestable metaphysical suppositions.

As such, today’s psychology of religion has limited itself to tasks like explaining religion as a form of ‘terror management’ in the face of mortality (31), in relation to personality traits, as a factor in moral decision-making, in terms of the human development trajectory, as a mental health asset, or as an epiphenomenon of a brain that has evolved to (over)detect agency (32). At the same time, contemporary religious studies has hewed toward the methods of sociology and anthropology, rather than psychology, to emphasize its object as a human social and cultural process (33), rather than a thing ‘given’ in ontology or a behavior to be understood in terms of neuro-psychological constructs (Though, it must be said that the cognitive science of religion has been forecast as a major growth area among certain quarters of the discipline (34)). For its part, the field of psychedelic studies was curtailed by the Controlled Substance Act of the late 1960s, just as it was beginning to burgeon. So, rather than being able to reconcile with its own esoteric roots, psychedelic science has re-emerged in the 21st century in a state of arrested development. Today, it is still making use of pre-1960s models of mystical experience that developed when psychology and religion were still intertwined with their own esoteric influences, rather than bringing together current religious studies research into its psychologized account of psychedelic experience. Thus dominated by the brain and mind sciences, psychedelic studies miss important tools from religious studies for characterizing non-ordinary experiences that get characterized as ‘mystical,’ and for working with psychonauts’ claims that these experiences afford “insights into the true nature of reality.”

### 2.2. A critical history of the scientific investigation of psychedelic-induced mystical experiences

The systematic investigation of phenomena deemed ‘mystical’ in the Western context emerged with the work of pioneering psychologist and philosopher William James (35). At the turn of the 20th century, he described a mystical experience as a unitary phenomenon that has four general qualities: ineffability, transiency, passivity, and a noetic quality (i.e., a sense of epistemological authority; (35)). James also introduced the notion that such experiences could be drug-induced—in his case, through the inhalation of nitrous oxide (35). Yet, while James still retains relevance in the study of religion, there were a whole host of other esoteric-science-religion thinkers that were relegated to ‘the dustbin of history’ in academia, despite achieving some staying power in today’s psychedelic research. A notable example is the British novelist and essayist Aldous Huxley. With his 1954 book, ‘The Doors of Perception’, Huxley was arguably first to widely popularize the concept that psychedelics could induce ‘mystical’-type experiences. Like other esoteric thinkers of the day, including Eveylnn Underhill and Mircea Eliade in the study of religion, and Carl Jung in psychology, Huxley had strong leanings towards Eastern and Western mystical traditions—as evidenced, for example, by the themes of his later novels such as ‘Time Must Have a Stop’ and ‘Island’, and his involvement in the Vedanta Society of Southern California. He even described his experience with the psychedelic compound mescaline in terms explicitly drawn from such traditions and went so far as to refer to psychedelics as “stimulators of the mystical faculties” (36). And, much like other esoteric thinkers of religion of the time, Huxley viewed mystical experiences in perennialist terms: as a discrete and unmediated (i.e., by conceptual frameworks) experience with phenomenological characteristics that cut across cultural and linguistic divides (37). In the present context, it is important to point out that it is unclear to what extent Huxley’s intellectual propensities (i.e., his ‘set’) played an active role in generating an experience that aligned with his conception of the mystical, and/or in biasing him towards interpreting an ambiguous experience as such. In addition, given the wide readership of ‘Doors of Perception’, this mystical framing likely influenced the collective ‘set’ of the many psychedelic users in the decade following its publication—thereby increasing their likelihood of experiencing putative mystical-like phenomena (and interpreting them as such) in a perpetual cultural feedback loop (38, 39).

Thinkers like James, Huxley, Underhill, Eliade, and Jung were key voices in the conversations regarding the possibility of creating a comparative study of mysticism. Arguably the most influential figure in approaches to the study of psychedelic-induced mystical experiences, however, was the philosopher William Stace. Stace was an English-born philosopher who grew up in a military family and eventually worked in the civil service, which is significant, because it was such Western European travel among the peoples they colonized that propelled speculation about a ‘common core’ underlying their different religions. This antiquated perspective, known as perennialism (33), dominates the way religion-related phenomena, like ‘mystical experience,’ are dealt with in psychedelic science today. The concept of ‘mysticism’ that Stace developed represents an elaborated version of James’ model and formed the primary basis of the ‘Mystical Consciousness Typology’—the first psychometric assessment of mystical-type effects, introduced by Walter Pahnke in 1963—which itself is the initial predecessor of the MEQ (see Table 1 for the factors that comprised this initial assessment, as well as other assessments of mystical effects). It is critical to note that, given their initial genesis and motivations, measures based on Stace’s work import his colonial, perennialist assumptions about ‘religion.’ As Taves (30) writes, “the theory underlying the ‘mysticism construct’ reflects a century of debate over the relationship between mystical, religious, psychotic, and drug-induced experiences that was fueled by an effort to identify the distinctive features—the ‘common core’ that ostensibly unites the religions of the world—and at the same time to defend the claim that religions provide access to ultimate reality.”

**Table 1:** The factor structure for each of the psychometric assessments used to measure mystical-type experiences as induced by psychedelic drugs.

Factors	Mystical consciousness typology (40)	MEQ-45 (41, 42)	MEQ-30 (43)	Hood mysticism scale (44)	11D-ASC (45)
Factor 1	Internal and external unity	Internal unity	Mystical	Unifying quality	Experience of unity
Factor 2	Positive mood	External unity	Positive mood	Positive affect	Spiritual experience
Factor 3	Transcendence of time and space	Deeply-felt positive mood	Transcendence of time and space	Temporal/spatial quality	Blissful state
Factor 4	Alleged ineffability	Transcendence of time and space	Ineffability	Ineffability	Disembodiment
Factor 5	Paradoxicality	Ineffability and paradoxicality		Noetic quality	
Factor 6	Noetic quality	Sense of sacredness		Religious quality	
Factor 7	Sense of sacredness	Noetic quality		Inner subjective quality	
Factor 8	Transiency			Ego quality	

Stace’s work, along with the theories and concepts of the other esoteric thinkers served as motivation for what is now known as the ‘Good Friday Experiment’, which occurred at Harvard University in the early 1960s under the supervision of the controversial figures Timothy Leary and Richard Alpert (later known as Ram Dass). This is where the first psychometric assessment of so-called ‘mystical experiences’ appeared. Walter Pahnke, then a doctoral student, decided to test whether psilocybin, when administered to Harvard Divinity School students in a religious setting, can induce mystical experiences similar to those reported by saints and mystics (40). The religious setting in this case was a ritual gathering of people in a church with a Christian leader giving a talk (‘sermon’), on the occasion of a significant moment in the Christian liturgical calendar: Good Friday. This occasion is a poignant, sombre, and ultimately hopeful one for Christians because it commemorates the state execution of their key figure, Jesus. They interpret his capital punishment as a religious ‘martyrdom’ that promises the spiritual ‘salvation’ of all his followers in the form of an eternal afterlife. We describe the nature of this ritual here because it is an important factor in the set and setting of study participants having a psilocybin experience in a specific ritual container. We attempt to do this description in generic, second order terms precisely because of how easy it is for Westerners to take Christian concepts for granted, as has been done with the ‘mysticism’ concept in psychedelic research. Note: the distinction we are making here is between the ‘etic’ and the ‘emic’ [see, e.g., (46): ‘etic’ refers to a scholarly reconstruction of a concept (outsider language), whereas ‘emic’ refers to a folk term (insider language)].

Using the direct precursor to the MEQ—the ‘Mystical Consciousness Typology’ derived from Stace’s work in comparative mysticism—Pahnke’s study found that, indeed, psilocybin was able to induce subjective effects that were mystical in character. According to this study, 4 out of 10 experimental subjects reached the 60–70% level of completeness on all components of the questionnaire, indicating a ‘complete mystical experience’ as defined by Pahnke, whereas no placebo subjects reached this level (40). In addition, all psilocybin-receiving subjects scored significantly higher on all components of the mystical experience questionnaire relative to placebo subjects (40). This study, therefore, provided the first evidence that psilocybin can induce effects indistinguishable from experiences considered traditionally mystical by perennialist theologians and esoteric thinkers of the day. In addition, the experience resulted in lasting positive effects at a 6-month follow-up, as indicated by psilocybin subjects averaging 50% of the maximum score in the ‘positive attitudinal/behavioral changes’ category of the assessments, compared to 15% in the controls (47). Strikingly, similar scores were also maintained at a 24–27 year follow up (47). As described by Doblin (47): “the experimental subjects wrote that the experience helped them to resolve career decisions, recognize the arbitrariness of ego boundaries, increase their depth of faith, increase their appreciation of eternal life, deepen their sense of the meaning of Christ, and heighten their sense of joy and beauty.” As apparent in this quote, the participants of this study exhibited strong existing ties to Christianity. This relationship to Christianity, and its attendant worldview, values, and conception of what is deemed ‘religious’ or ‘spiritual’, constitute a critical part of the ‘set’ that primed them for the psychedelic experience they would have under the study circumstances (‘setting’).

Importantly, this set entails a worldview in which mystical experiences are positively valanced, as is spirituality. Thus, we can see that Pahnke’s investigation of mysticism was conceptually and experimentally embedded in a larger structure referred to as ‘religion,’ and since ‘religion’ is a category (an abstraction), practically speaking, Pahnke’s experiment had to take place within a *particular one*. Though Marsh Chapel is nondenominational, it was founded by a Methodist leader. Methodism was an 18th-century charismatic Protestant movement that valued direct personal experience of being sensuously overtaken by their god (in a form called ‘the Holy Spirit’), the embodied experience of a ‘strange warming of the heart,’ and revelations from their god. These kinds of experiences, then, are templated into the earliest ‘mystical experience’ assessment as markers of ‘true’ or ‘authentic’ mysticism.

But then where do experiences of contact with malevolent entities or experiences of going to ‘hell’ realms or experiences of being violently consumed or entirely fused with a nihilistic universe fit? These are certainly all well described in the trip reports documented in archives such as Erowid, a non-profit psychedelic education and harm-reduction website founded in 1995 which allows individuals to add written trip reports to a public repository. They do not fit in the rubrics of ‘mysticism’ or ‘spirituality’ as used in the psychedelic science literature because these concepts are always already defined as positive^1^. This is not a problem in and of itself, but only when the rationale for these assumptions is not laid out clearly. In the case of the definition of spirituality taken up in the psychological disciplines, we can see that it is often informed by data-driven approaches, where researchers ask particular communities what spirituality means to them. For instance, the millennial emerging adults studied by several researchers describe as ‘spiritual’ their experience of awe, belief, and interconnection (48, 49). This is therefore a particular emic concept of spirituality, which we must be very careful not to accidentally imply can stand for the thing itself, what the term ‘really’ means, or ought to mean, by failing to contextualize and historicize its cultural specificity. We would obtain a different answer about what types of experiences and things could be considered spiritual if we asked magicians that associated themselves with the controversial esotericist Aleister Crowley (for these esotericists, evil beings, forces, and realms would certainly qualify as spiritual things).

The perennialist and Christian biases of the earlier assessment persisted into the next generation of psychedelic research. This arguably occurred largely via psychedelic researcher Bill Richards, who had worked with Pahnke in the 1960s and who has now been involved in psychedelic research at Johns Hopkins University over the past two decades. Richards played a primary role in developing the MEQ-43, which was directly derived from the Mystical Consciousness Typology used in the original Pahnke study and highly influenced by Stace. This measure was used in a 2006 study at Johns Hopkins University which replicated the main results of the Good Friday Experiment (41). Distinct from the original study, psilocybin was now administered in a warm and supportive environment that featured minimal religious overtones apart from any implicit religious bias on behalf of the subjects themselves or the study’s administrators. Subjects were healthy individuals and were not selected for strong religious inclinations. This study found that psilocybin use could elicit mystical-type experiences as indexed by scores on the MEQ-43 and, moreover, that these experiences were rated as highly personally meaningful and as having lasting positive effects on mood and behavior (41, 50). Subsequent studies by this research team have further replicated these findings (19, 43). These latter studies were conducted in the context of refining, validating, and testing the MEQ (see Table 1 below for the factor structure of the different versions of the MEQ, as well as other related measures).

### 2.3. Christianity as the implicit paradigmatic example in western concepts of ‘mysticism’ and ‘religion’

For the reasons described above, we contend that the concept of ‘mysticism’ that was originally taken up in psychedelic research is fraught with limitations and intrinsic biases that are seldom acknowledged in the field (see (30) for more discussion). One aspect that warrants additional emphasis is that, since there is frequent reference to ‘mysticism’ being a function of religions, those of us engaged in psychedelic research must acknowledge the set of assumptions involved in the very concept of ‘religion’. Indeed, it is important to recognize that the concept of ‘religion’ as it is defined in the English language, takes Christianity as its paradigmatic example (see (51) and (33) as entry points to the vast literature on this issue).

The reason that Christianity has been *the* model of religion has a deep history. In the first place it owes to the fact that Christianity became a hegemonic political force in the Western world when the Roman Empire took up its mantle in classical antiquity. Thus, when the medieval and then the modern university system developed in Europe, it was Christian worldviews and values it sought to explain and uphold (33). Following this pattern, North American universities typically grew up around a Christian theological school. Because of the colonial encounters taking place in the ‘Age of Discovery’ (~ 15th–17th century CE), Christianity was not only the main object of study in universities, but the paradigm case through which scholars understood all other behaviors and artefacts deemed ‘religious’. Essentially, whenever European colonizers saw people doing anything that looked like *their* Christian religion, they construed it as a part of those people’s religion. Colonizers largely ignored or persecuted what did not look like their own Christian behaviors and artefacts (e.g., bibles, cathedrals, and men in robes holding forth), dismissing other lifeways as “primitive” to the extent that they did not categorically separate out “religion” from the activities of everyday living, and attested instead to perceiving everything as sacred and integrated. It was thus that the ‘world religions paradigm’ was born, with Christianity at the civilizational top, the other four of the ‘big five’ (Judaism, Islam, Hinduism, and Buddhism) set underneath it, and the ‘primitive’ religions of Indigenous peoples at the bottom (e.g., (52)).

In the mid-20th century, academic theology finally splintered under the pressure of the burgeoning disciplines of the social sciences, introducing a new *secular* discipline of religious studies to the academy, in addition to the existing array of academic programs in theology (53). Religious studies, being based on the academic model of Christian theological schools, remained Christian-centric until major anticolonial shifts began taking place in universities over the past 20 years. “Mysticism” is a concept that has been updated in this shift within the discipline of religious studies, but, as we observe, not within others, like the psy disciplines, who sometimes use it. For this reason, the historicization and cultural analysis of religion-related terms (i.e., like ‘mysticism’) is essential to clarify implicit, deeply rooted biases.

### 2.4. Section summary

As described, the creation and refinement of psychometric assessments of mystical experience have occurred in the context of colonial, perennialist, and Christian-Protestant perspectives. In order words, they have been motivated by a view of mystical experience as a fundamentally positive, sui generis phenomenon that lies at the heart of diverse traditions, cutting across cultural and linguistic divides. This is particularly through the influence of Walter Stace in the generation of psychometric assessments of mystical experience, as well as the disciplinary disconnect between the psychological sciences and critical work in the humanities and other social sciences. Indeed, it is evident that, although they were initially derived from early scholarship on comparative mysticism, contemporary measures of mystical experiences (e.g., the MEQ) and the research literature in which they are embedded only loosely interface with contemporary scholarship in disciplines that are responsible for historical and cultural work, such as religious studies, philosophy, and anthropology. As a result, research on psychedelically-induced mystical experiences arguably lacks historical and cultural context, as well as awareness of the intrinsic limitations and biases of its current conceptualizations and assessments. More specifically, psychedelic research has only minimally engaged with work indicating the unstable and culturally local nature of concepts such as ‘mysticism’ and ‘religion’, or with work indicating that the perennialist project of trying to find a ‘common core’ of religion is in fact heavily biased by a Euro-American Christian/Protestant framework.

Moreover, it is evident that the beliefs, assumptions, and frameworks articulated by influential thinkers such as Stace and Huxley around the midpoint of the 20th century made a significant impression upon the psychedelic culture at large, thereby likely influencing their ‘set’ and therefore the nature of their psychedelic experiences. This suggests that a variety of—typically relatively unacknowledged—cultural influences and feedback loops have given rise to the assessments and conceptualization of psychedelic mystical experiences that are now taken to be the status quo. We believe that greater explication and acknowledgement of such influences is essential for creating more refined and culturally-sensitive measures of psychedelically-induced mystical-type phenomena. Next, prior to describing some forward-looking suggestions on how to accomplish this, we briefly comment on a recent discussion of whether ‘mystical experience’ should be seen as a construct suitable for empirical research at all.

## 3. Ambivalence towards the mystical in contemporary psychedelic research

Researchers in the emerging field of psychedelic science have voiced some ambivalence towards the inclusion of ostensibly ‘non-scientific’ language (i.e., such as relating to ‘mystical experiences’) in psychedelic research. Notably, Sanders and Zijlmans (26) recently called for the field of psychedelic science to “move past mysticism” entirely. For them, the risks and difficulties of using a “framework associated with supernatural or nonempirical belief systems” are too great, and the only solution is to ‘demystify’ our model of the psychedelic state (26). We contend that this perspective is a direct consequence of the contemporary siloing, discussed above, of scientific research on psychedelic-induced mystical experiences from existing research on mystical/spiritual/religious type phenomena—whether in the humanities (30), or even within other subfields in psychology and neuroscience (54–57). We agree with the two responses to the above-mentioned article (58, 59), which collectively highlight that mystical experiences are, in principle, valid and operationalizable objects of scientific inquiry with a rich history as such (within and outside of psychedelic research), and that their empirical investigation is fundamentally independent of the metaphysical claims derived therefrom (also see (60)). We further point out that the tendency for mystical experiences to be interpreted as having metaphysical or spiritual significance is itself an interesting and valid topic of empirical investigation. This is exemplified, for example, by a recent study which found that psychedelic use can lead to changes in metaphysical beliefs, with a bias away from physicalism towards greater endorsement of panpsychism and mind–body dualism (61).

We emphasize here that, although we have critiqued and drew attention to the limitations of existing conceptualizations and assessments of mystical experience, we are not arguing for a wholesale removal of the term ‘mystical’ and related concepts and frameworks from psychedelic science. Rather, we believe that if they are used, then greater attention should be paid to the implicit assumptions and biases inherent in their current usage, and that alternative and more refined and culturally-sensitive assessments should be devised which afford a broader operationalization of such phenomena that is aligned with relevant interdisciplinary scholarship.

The above points notwithstanding, we agree with Sanders and Zijlmans that over-reliance on recourse to ‘mystical-type’ effects as a means of measuring this particular aspect of psychedelic subjective effects has its limitations. The creation of a statistically validated and reliable psychometric assessment does not necessarily entail the ontological status of its purported referent as a discrete, *unified* phenomenon. Rather—as also touched upon by Sanders and Zijlmans—in the absence of a clear and robust causal mapping between the purported referent and (neuro)biology, the assessment simply serves as a heuristic tool to assess a set of ostensibly interrelated and temporally co-occurring set of effects—which may very well be better captured by a different set of abstractions. As an additional example, a similar state of affairs is the case for the personality construct of ‘absorption’ (62). Absorption has a multi-decade history of investigation and has been found to reliably correlate with a variety of traits and behaviors, including sensitivity to psychedelic drug effects (63). Yet, ongoing debate exists as to whether absorption should be understood as a single trait or as a heterogenous bundle of tendencies and predispositions that often co-occur (62, 63). In other words, the construct of ‘absorption’ as measured by the Tellegen Absorption Scale—in a similar fashion to the construct of ‘mystical experience’ as measured by the MEQ—appears to have strong reliability and heuristic utility, but unclear construct validity. In both cases, exploring alternative conceptualizations through targeted construct validation research is required to ascertain whether they are indeed assessing a discrete and unified psycho-neurobiological phenomenon. As described in Section 4.1 below, we believe that complementary attempts at conceptualizing the subjective phenomena that occur in so-called mystical experiences in terms of existing psychological or neurobiological constructs may make such experiences less of a ‘black box’ and more amenable to rigorous empirical investigation and construct validation.

## 4. Advancing theoretical perspectives and empirical approaches to psychedelic-induced mystical experience

So far, we have offered a historical/cultural critique of conceptualizations and psychometric assessments of mystical experiences in the context of psychedelic research. In this final section, we offer some forward-looking suggestions on ways in which this area can be fruitfully advanced. In particular, we suggest two complementary approaches to overcoming previous limitations. The first is to seek to decompose the complex phenomenon of ‘mystical experience’ into empirically-supported constructs drawn from psychology and neuroscience, thereby ‘demystifying’ it, absolving it of its ostensible *sui generis* status, and situating it within existing research literature. We emphasize that this is not meant to deny the existence of something called a ‘mystical experience’, but, rather, to offer an alternative lens on the experience that may have pragmatic utility in the context of scientific research. Second, we draw on the work of religious studies scholar Ann Taves to highlight the value of cross-cultural sensitivity, item-level validation, and narrative reports for coming to more refined and nuanced assessments of mystical experiences.

### 4.1. Decomposing the mystical experience into psychological and neural constructs

It is important to acknowledge that, at a fundamental level, so-called ‘mystical experiences’ are complex subjective experiences that are comprised of changes spanning cognition, emotion, perception, and sense of self. Chief among these are changes to one’s sense of self or ego, which feature predominantly in experiences of unity, which have been construed as the hallmark of mystical experiences as assessed in psychedelic research. In addition, given that psychometric ratings of such experiences are continuous rather than discrete, and given that factor analyses have revealed the statistical dissociability of their component parts, it is likely that several distinct neuro-psychological functions are at play which can differ in their relative degree of occurrence at any given moment, or in a given instance of such an experience. An important question with regard to rigorously characterizing these experiences, therefore, is: of what distinct neuro-psychological constructs are they comprised?

**Figure 1:**
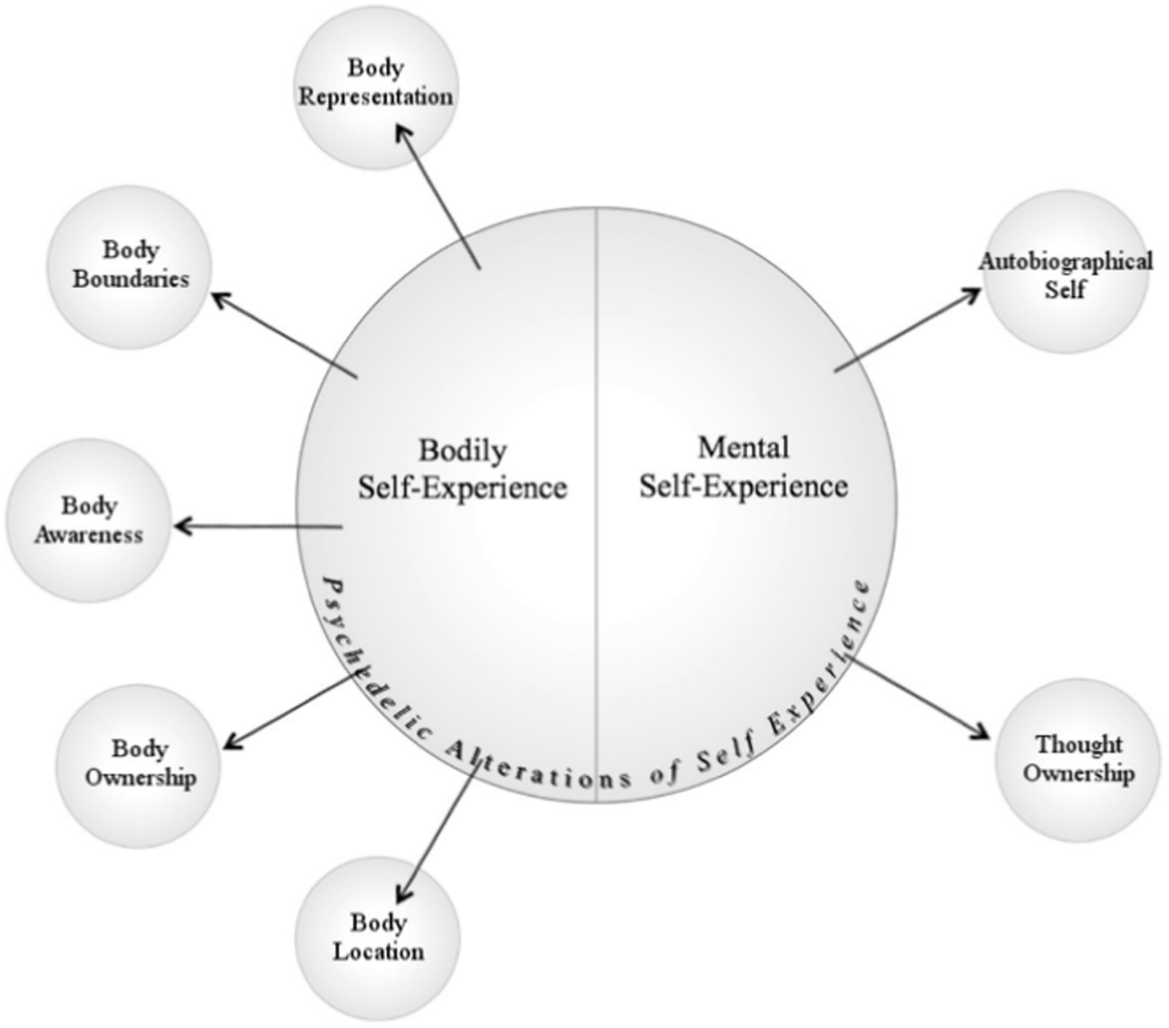
Components of self-experience altered by psychedelics. Adapted from (64).

Towards this end, Girn and Christoff (64) separated psychedelic alterations of self-experience—which are, as mentioned, putatively central to mystical-type effects—into the two categories of ‘bodily self-experience’ and ‘mental self-experience’. Within each of these categories, they list constructs from cognitive psychology/neuroscience that psychedelics putatively alter in the context of mystical-type experiences and more generally (Figure 1). Critically, each of these proposed components have their basis in well-characterized research literatures that are independent of psychedelics (see (64); Table 1). Accordingly, this proposed taxonomy was explicitly aimed at conceptualizing these alterations in generic, second-order terms that can bridge contemporary cognitive neuroscience and research on psychedelics.

As highlighted by Girn and Christoff, this conceptualization of psychedelic effects affords a view of psychedelics as valuable experimental tools to study the usually seamlessly integrated components of selfhood, in a manner analogous to the way sensory mismatch paradigms have been used to identify the neural bases of certain types of bodily self-experience [e.g., (65)]. Given the centrality of self-related changes in mystical-type experiences, this lens could be applied to empirically ‘demystify’ mystical-type phenomena and facilitate deeper understanding of their neuro-psychological basis.

This taxonomy dovetails nicely with the distinction between the ‘narrative’ and ‘minimal’ self, which was initially developed in work in the philosophy of mind (66), and then ported to cognitive neuroscience (67, 68) and, more recently, used to describe psychedelic effects (69). The minimal self represents the bare sense of inhabiting a first-person perspective—the sense of ‘I am’ (66). It is viewed as a fundamental property of selfhood that is predicated on interoceptive and viscerosomatic inputs which combine to give a sense of existing from a particular spatial location (i.e., in ‘this’ body). Scaffolded on this, is the narrative self (also referred to as the autobiographical self). This higher-order aspect of selfhood represents our conceptually-based identity which consists of traits, self-beliefs, and personal characteristics, and which is embedded within a temporally extended narrative linking memories of the past to an imagined future (66, 68). Unitive mystical-type experiences seem to imply a dissolution of either one or both of these types of self, however this has not been directly investigated. Employing the taxonomy of Girn and Christoff and/or this distinction between two primary forms of self has strong potential to advance our empirical understanding of mystical experiences.

In 2016, Matthew Nour and colleagues proposed and validated a novel assessment of psychedelic effects, referred to as the Ego Dissolution Inventory (70). This assessment seeks to measure experiences induced by psychedelics in which boundaries between self and world were blurred or dissolved entirely, without invoking the language of mystical experiences. In this way, it represents an alternative metric to assess a putatively core component of mystical experience, without necessarily carrying limitations and connotations of the preceding mysticism-focused instruments. However, this measure is still notably limited in that it does not feature explicit linkages to existing psychological constructs of the self and is thereby relatively siloed from research literatures that have significant potential to enrich its understanding.

Providing a complement to quantitative assessments of psychedelic effects, studies have also analyzed patient responses to questions in structured interviews. A relevant common theme reported by patients in these studies is that of ‘connectedness’—a concept also intimately connected to the unitive aspect of mystical experiences as assessed by existing measures (71, 72). Investigations with the psychedelic brew ayahuasca revealed experiences of connectedness to be of a tripartite character—separable into connection with self, connection with others, and connection with spirit or nature (73–75). This aligns strongly with the reports of patients who received psilocybin-assisted therapy for treatment-resistant depression, who reported moving from feelings of disconnection from self, other, and world to feelings of connection in these three domains (71). As a result of the centrality of feelings of connectedness, a novel scale called the ‘Watts Connectedness Scale’ (WCS) was recently developed (76). This scale captures elements of post-acute psychedelic experience that are highly related to the acute effects targeted by the MEQ, but does so in non-mystical language. Given that his scale was developed based on clinical findings with a goal of isolating therapeutically-relevant outcomes, it is arguably in itself not ideal for gaining deeper empirical understanding of acute experiences. However, it again points to the ways in which mystical experiences may be valuably reconstrued.

It is important to point out that scores on the MEQ, the ‘Oceanic Boundlessness’ subscale of the 5D-ASC, and the ego-dissolution inventory are highly positively correlated. Psychometrically, it may be arguable that each of these measures differ in label alone and, in reality, index the same underlying construct. However, it is critical to highlight that different labels give rise to distinct connotations and semantic associations that can impact their interpretation and their perceived relatedness to other concepts and constructs. As described above, the language of the MEQ gives rise to associations with religion, thereby implicitly linking the measured phenomenon with related discourses and bodies of work. In contrast, the labels of ‘oceanic boundlessness’ and ‘ego-dissolution’ may give rise to associations with Freudian concepts, which in turn also facilitates linkages to distinct literatures. In the present work, we are highlighting concerns specific to the use of the language of ‘mysticism’, but do not discount concerns related to other conceptualizations.

With concepts like ‘ego dissolution’, ‘connectedness’, and a taxonomy of alterations of self-experience in hand, we can ask more fine-grained questions about psychedelic experiences: are positive and negative ego-dissolution experiences related to different patterns of changes in the sub-types of mental and bodily self-experience? Can we experimentally manipulate these patterns by altering the ‘set and setting’ or incorporating behavioral paradigms? Can novel molecules be designed to have and not have certain effects? The consequences of investigating (or not investigating) these questions are particularly relevant insofar as ‘mystical experience’ or ‘ego dissolution’ is cited as a primary mechanism in psychedelic-assisted psychotherapy.

At the same time, we must take this decomposition of ‘mystical’ type experience even further by asking: how do some of these models reify problematic mind/body dualism, given that many non-Cartesian worldviews show us that there is no possibility of an autobiographical memory without a body, and there is no bodily experience without a mental representation and interpretation? Are there yet more ways to break down this model of mental and bodily self-experience that do not assume Western European models of consciousness so prevalent throughout neuroscientific and psychedelic discourse? In fact, this is where we would do well to heuristically try out models of self, self-other relationship, etc. from worldviews that are not dominant in the academy, especially from the holistic and process-based models found in many of the Indigenous cultures from which psychedelic substances and practices are being appropriated. These models are worthwhile candidates for use in psychedelic research, with their metaphysical assumptions and cultural locatedness, just as Hood, Stace, and Pahnke’s models were with theirs.

For instance, anthropologist Colin Scott (77) writes:

In Cree, there is no word corresponding to our term “nature.” There is a word pimaatisiiwin (life), which includes human as well as animal “persons.” The word for “person,” iiyiyuu, can itself be glossed as “he lives.” Humans, animals, spirits, and several geophysical agents are perceived to have qualities of personhood. All persons engage in a reciprocally communicative reality. Human persons are not set over and against a material context of inert nature, but rather are one species of person in a network of reciprocating persons. These reciprocative interactions constitute the events of experience.

Though we cannot explore the question in depth within the remit of this paper, we can ask: how does ego-dissolution or unitive experience function for people who already inhabit a worldview where everything is experienced as having a person-like animacy and as being already united in a whole comprised not of discrete nouns, but by the *process of living*?

In the next section we explore an ongoing attempt at producing cross-culturally relevant psychometric scales for psychedelic experience, and possible future directions for doing so.

### 4.2. Cultural sensitivity and the need for psychometric meta-data

In 2020 the religion scholar Ann Taves made a vital contribution to dealing with ‘mysticism’ in psychedelic science and research on other non-ordinary states of consciousness (30). Recognizing that the existing tools (described above) named their factors after descriptions of non-ordinary experience worded in the insider terms of particular religious communities, or the metaphysics of prominent esoteric thinkers, she sought to redescribe them in generic, scholarly terms. She thus developed the Inventory of Non-Ordinary Experiences (INOE), whose goal was to test the possibility for creating generic psychometric items that can be recognized across cultures, including religious cultures, national cultures, linguistic cultures, and so on. To be clear, Taves is not making the neo-perennialist case that these words or concepts are at the basis of all non-ordinary experiences, but, rather, seeking words that are interoperable between cultures as an attempt to build linguistic bridges to facilitate understanding.

As a starting point, Taves and her team created a list of approximately 75 items, many of which were extracted from existing measures, including the above-mentioned mysticism scales, the Appraisals of Anomalous Experience Interview [AANEX; (78)], which is a measure of psychological responses to anomalies associated with psychosis, and the Survey of Anomalous Experiences [SAE; (79)], which is a questionnaire querying how people attribute unusual experiences (specifically ‘parapsychological’) to paranormal agents. In order to generate generic factors—that is, factors which describe experiences independent of particular culturally-based valenced appraisals—questions related to the occurrence of a particular experience were distinct from questions pertaining to the experience’s origin, long-term effects, context, balance, frequency, and significance. In addition, Taves and colleagues’ approach depended heavily on item-level validation *via* the collection of psychometric meta-data. In this context, ‘psychometric meta-data’ refers to responses to questions *about* the questionnaire items. For each item, participants were asked to paraphrase the item in their own words, provide details on how they would respond, and give an actual or hypothetical example of the experience referred to by that item (30). This meta-data provided valuable information on whether each item was understood consistently—or understood at all—and enabled iterative refinement of the wording used. Notably, this validation approach was conducted with independent samples in the United States and India, in English and Hindi, respectively. This revealed significant differences in interpretations of items across cultures and the need for distinct wordings to convey similar concepts. Moreover, this validation procedure revealed that many of the items, including those drawn from widely-used mysticism scales, were inconsistently interpreted across participants and required multiple iterations of refinement to more uniformly convey the intended meaning (30). This suggests that participants may be routinely responding to such items in idiosyncratic ways, highlighting significant limitations in their application and interpretation. More refined assessments of mystical experiences, therefore, would do well to collect psychometric data to best ensure uniformity in item interpretation and alignment with the intended phenomena of interest for the target demographic in question.

Furthermore, the collection of qualitative meta-data such as described above underscores the value of narrative reports. Such reports are essential in order to gauge idiosyncratic appraisals and construals of similar types of experiences, and how these may vary across cultures and influence responses to quantitative psychometric instruments. We suggest that, in the context of mystical experiences, narrative self-report measures should not only be included, but should also be structured so that respondents are encouraged to make fine-grained distinctions between alterations in different aspects of sense of self, and describe the sensorial, emotional, and cognitive experiences of these alterations. This could be supported or complemented by microphenomenological interviewing (69, 80), where a researcher guides the recall of an experience and the participant’s reflection on different aspects of it.

Structured narrative reports such as these have potential to facilitate the refinement of psychedelic assessments that separate out different components of the experience, yielding further fine-grained data. For instance, ‘ego-dissolution,’ as described above, has gained increasing traction as a more secular, second order concept underlying the ‘mystical’ one, but it is perhaps only one among others. Narrative reports may suggest querying aspects of psychedelic experience that have previously been ignored by existing scales, given their focus on either a religious type of mysticism or a psychopathological type of psychosis. Conducting this type of qualitative research requires cooperation between psychedelic scientists and humanists and social scientists who are aware of the cultural history of ideas like ‘mysticism,’ or ‘spirituality,’ or even ‘ego,’ and have a sophisticated theoretical model of religion that views it as provisional human concept that can be used to analyze cultural processes, not a sui generis reality that is ‘out there.’

Psychedelic scientists and humanists should also explore more ways of relating ‘naturalistic’ psychedelic experiences to the experiences of trial participants in clinical settings, using a mix of quantitative and qualitative methods. Some recent contributions from medical anthropologist Olivia Marcus (81–83) and multidisciplinary scholar David Yaden (21, 84) offer excellent models for this type of work. Marcus’ long-term fieldwork at a psychedelic retreat center in the Peruvian Amazon investigates what she calls ‘therapeutic pluralism’ in the treatment of mental health conditions. Her research tracks the dialog among shamans, mental health practitioners, and their clients with respect to their ayahuasca use. At the same time, with a team of clinicians and research scientists, she has also used her ethnographic findings to inform clinical tools, namely, a protocol for outcome evaluation of ayahuasca-assisted addiction treatment. Similarly, Yaden has used his combined knowledge of non-ordinary consciousness from religious studies and from psychology to show how the study of rituals in different religions can inform research and clinical contexts. The point of such work is neither to affirm that any particular religion got things ‘right,’ nor to ‘explain away’ religion as a neuro-cognitive epiphenomenon, but rather to make comparisons between psychedelic experiences and other non-ordinary states. Making these comparisons can do two crucial things: (1) give us clues about the specificity of what psychedelic *molecules* are doing; and (2) build more robust models of what psychedelic *experiences* are doing. There is no way to do this work but in research collaborations between experts in comparative religion, anthropology, sociology, philosophy, and psychology, cognitive science, and neuroscience.

## 5. General discussion and conclusions

### 5.1. Moving forward with ‘mysticism’ in mind

The foregoing sections have demonstrated the need for a number of different lines of research between psychedelic science and humanities stemming from the ‘mysticism’ concept. There is the need to historicize the language of ‘mysticism’ and other religion-relevant concepts (like ‘spirituality,’ ‘sacred,’ ‘divine,’ etc.) as they emerge from religious, Indigenous, underground psychonaut communities, and scientific networks. There is also a need for an ongoing historicizing of the psychedelic present by critically analyzing the current discourse among all psychedelic stakeholders, and to track relationships between the unlikely bedfellows in the contemporary psychedelics space. That is, we need philosophical and anthropological research which is dedicated to dialoguing with the scientists and clinicians actively using these concepts both in their scholarly research and ‘behind the scenes’ of conference presentations and publications. These dialogues should query their strategies, motivations, and beliefs about the types of ‘mystical’ (etc.) experiences possible in scientific and clinical studies and beyond. There is also tightly focused psychometric work to be done in refining psychedelic assessments that can help provide a more accurate representation of people’s non-ordinary experiences.

These efforts will set the stage for future work on potential pathways to bridge psychedelic science and humanities scholarship, creating the grounds for consilience between these still-disparate domains and contributing to the cultural containers that will allow more thoughtful public consumption and understanding. Consilient psychedelic theory will benefit the public discussions that are underway in the current psychedelic moment, including issues of decriminalization and legalization, accessibility and corporatization, medicalization and cognitive liberty, and the lines between cross-cultural learning and colonial appropriation.

### 5.2. Conclusion

In this paper we have argued that research on psychedelic-induced mystical experiences exhibits a number of limitations and biases which are a result of a lack of intersection between psychedelic science and contemporary scholarship on mysticism and other religion-related concepts from religious studies and cognate disciplines. This scholarship shows the mysticism concept to be fraught with metaphysical assumptions and cultural biases which have not been sufficiently recognized or reckoned with in the psychedelic science literature. Our core contention has been that, if ‘mysticism’ is used, then researchers must begin to do this reckoning and/or pursue alternative ‘non-mystical’ conceptualizations of psychedelic effects, still with attention to operationalizing these alternative concepts in culturally sensitive and properly historicized ways. In sum, we have sought to display how interdisciplinary psychedelic scholarship can offer a more nuanced, cross-contextually relevant, and empirically rigorous approach to studying psychedelic experiences.

## Author contributions

SM and MG conceived and of the idea and wrote the first draft. LR provided edits and suggestions, and assisted with draft finalization. All authors contributed to the article and approved the submitted version.

## Conflict of interest

The authors declare that the research was conducted in the absence of any commercial or financial relationships that could be construed as a potential conflict of interest.

## Publisher’s note

All claims expressed in this article are solely those of the authors and do not necessarily represent those of their affiliated organizations, or those of the publisher, the editors and the reviewers. Any product that may be evaluated in this article, or claim that may be made by its manufacturer, is not guaranteed or endorsed by the publisher.

## Footnotes

^1^An additional psychometric assessment commonly used in psychedelic research—the five-dimensional altered states of consciousness questionnaire (5D-ASC)—features a factor (‘Oceanic Boundlessness’) that correlates highly with the MEQ. It also has a factor (‘Dread of Ego Dissolution’) that loosely pertains to what might be viewed as ‘negative’ mystical-type experiences. This measure, however, does not explicitly claim to assess mystical-type phenomena and has been less examined in clinical research. Oceanic boundlessness, coming from Freudian depth psychology, has its own religious and cultural biases, some of which overlap with the esoteric roots of the mysticism concept.
